# Clinico-Epidemiological Study on Cerebral Venous Sinus Thrombosis: Manifestations, Complications, Risk Factors, and Diagnostic Challenges

**DOI:** 10.7759/cureus.102448

**Published:** 2026-01-27

**Authors:** Shatha S Abdulaziz, Ali R Hashim, Nazik H Hasrat, Hassan A Farid

**Affiliations:** 1 Internal Medicine, Basrah Teaching Hospital, Basrah, IRQ; 2 Medicine, College of Medicine, University of Basrah, Basrah, IRQ; 3 Community Medicine, Basrah Health Directorate, Basrah, IRQ; 4 Neurology, Manchester Centre for Clinical Neurosciences, Manchester, GBR

**Keywords:** cavernous sinus, cvst, hypercoagulability, sagittal sinus, thrombosis, transverse sinus

## Abstract

Cerebral venous sinus thrombosis (CVST) is a rare but potentially serious cerebrovascular disorder caused by thrombosis of the cerebral venous sinuses, which can result in raised intracranial pressure, cerebral edema, and hemorrhagic complications. The clinical presentation is highly variable, often leading to diagnostic delays despite advances in neuroimaging. This study aimed to describe the clinical, demographic, and epidemiological characteristics of patients with CVST, identify associated risk factors and comorbidities, and evaluate complications and long-term outcomes. In addition, the study sought to assess diagnostic practices and challenges in the detection of CVST. This single-center cross-sectional study included 21 patients with radiologically confirmed CVST who presented to the Neurology Unit at Basra Teaching Hospital between January 2024 and January 2025. Clinical, demographic, imaging, and outcome data were retrospectively collected and analyzed. The mean age of the patients was 37 years (range: 14-95 years), with a predominance of women (n = 13, 61.9%). The most common risk factors identified were oral contraceptive use and infections (n = 7, 33.3% each), followed by obesity (n = 5, 23.8%), smoking (n = 5, 23.8%), and pregnancy/postpartum state (n = 4, 19%). The most frequent presenting symptom was headache (n = 15, 71.4%), followed by seizures and visual disturbances (n = 9, 42.9% each). The sagittal sinus was the most common site of thrombosis (n = 9, 42.9%), followed by the cavernous sinus (n = 8, 38.1%) and the transverse/sigmoid sinus (n = 4, 19.0%). Diagnostic challenges were prevalent, with non-specific symptoms (n = 12, 57.1%), delayed presentation (n = 11, 52.4%), and limitations of imaging techniques (n = 9, 42.9%) contributing to late diagnosis. Regarding treatment, all patients received symptomatic management, while anticoagulation therapy was administered to 95.2% (n = 20). Immediate complications included acute seizures (n = 10, 47.6%) and intracranial hemorrhage (n = 6, 19.0%). Persistent headaches (n = 13, 61.9%) and epilepsy (n = 6, 28.6%) were the most frequent long-term complications. Statistical analysis revealed no significant association between demographic factors, risk factors, or clinical features with the type of venous sinus thrombosis. In conclusion, CVST predominantly affects young to middle-aged women, with hormonal factors and infections being major contributors. The diagnosis remains challenging due to non-specific symptoms and imaging limitations, often leading to delays in treatment. Despite advances in management, CVST is associated with significant immediate and long-term complications, emphasizing the need for early recognition and intervention. Improved awareness, risk factor identification, and timely imaging are crucial to enhancing outcomes in CVST patients.

## Introduction

Cerebral venous sinus thrombosis (CVST) is a rare but serious neurological disorder with high morbidity and mortality if diagnosis and treatment are delayed. Its incidence is estimated at three to four cases per million annually, with a higher prevalence in women of reproductive age due to hormonal factors such as oral contraceptive use, pregnancy, and puerperium [1,2]. Clinical presentation is diverse, ranging from headache, seizures, and focal neurological deficits to intracranial hypertension and coma [3,4]. Delays in diagnosis are common, with median intervals of several days between symptom onset and hospital presentation [4].

Multiple risk factors often contribute to CVST, though approximately 15% of patients present without an identifiable cause. Hormonal factors, genetic thrombophilia, and chronic inflammatory or autoimmune disorders are major contributors [3,5]. Infections such as otitis media and mastoiditis, as well as head trauma, jugular vein catheterization, and lumbar puncture, may also trigger venous thrombosis [6]. Overall, most cases involve a prothrombotic state with either inherited or acquired predispositions.

The pathophysiology of CVST is characterized by venous outflow obstruction leading to increased venous and capillary pressure, reduced cerebral perfusion, and subsequent cerebral ischemia. Cytotoxic and vasogenic edema develop, and in severe cases, intracerebral hemorrhage (ICH) occurs [3,7]. Persistent thrombosis may block cerebrospinal fluid absorption at the arachnoid granulations, raising intracranial pressure and worsening neurological outcomes [7].

CVST presents highly variable and often non-specific symptoms, complicating diagnosis. Headache is the most frequent symptom, occurring in up to 90% of patients, and may mimic migraine but typically persists and worsens over days or weeks. Intracranial hypertension can exacerbate pain during coughing or Valsalva maneuvers and may cause visual disturbances, diplopia, or papilledema [4]. Seizures occur in about 40% of cases, often focal and at times generalized, while focal neurological deficits, including hemiparesis and aphasia, are also common. Severe cases involving the straight sinus or large venous infarcts carry a high risk of coma and death [8].

Rapid diagnosis is essential for preventing complications. Neuroimaging is the cornerstone of diagnosis, with CT and MRI/magnetic resonance venography (MRV) the most commonly used modalities. CT may reveal hyperdense venous sinuses, venous infarction, or hemorrhage, with the classic “empty delta sign” appearing on contrast studies. MRI with MRV is more sensitive and specific, showing thrombus-related signal changes and venous flow defects [3,6]. D-dimer assays, while helpful, are insufficient to rule out CVST on their own; neuroimaging is always required when clinical suspicion is high [7].

Management focuses first on stabilizing the patient by controlling seizures, reducing intracranial pressure, and preventing herniation. Anticoagulation with heparin, followed by transition to oral anticoagulants, is the mainstay of therapy, even in the presence of hemorrhage, as it promotes recanalization and prevents further thrombotic events [4]. In rare cases with deterioration despite anticoagulation, thrombolysis or surgical thrombectomy may be considered [8].

CVST is associated with increased intracranial pressure, seizures, hemorrhagic transformation, venous infarction, and long-term neurological deficits. Mortality is usually linked to herniation following massive thrombosis or hemorrhage. Early recognition and timely treatment are critical for reducing morbidity and improving outcomes [4].

The primary objective of this study was to characterize the clinical and demographic features of patients with CVST and to identify associated risk factors. Secondary objectives were to evaluate short- and long-term clinical outcomes and to assess current diagnostic practices, with particular emphasis on neuroimaging approaches and factors contributing to delayed diagnosis. Overall, the study aims to generate region-specific data to inform clinical practice and identify areas for improvement in the timely diagnosis and management of CVST.

## Materials and methods

This study was a cross-sectional observational study conducted at Basrah Teaching Hospital to assess the clinical manifestations, complications, risk factors, and diagnostic challenges in patients with CVST. Data were collected over a one-year period from January 1, 2024, to January 1, 2025.

All consecutive patients presenting to the Emergency Department or admitted to the Neurology Unit of Basrah Teaching Hospital during the study period with suspected CVST were screened for eligibility. Case ascertainment was based on consecutive Emergency Department presentations and inpatient neurology admissions to minimize selection bias. Patients were identified through neurology admission logs, Emergency Department records, and radiology databases.

Upon presentation to the Emergency Department, patients underwent a standardized initial clinical assessment, including a detailed neurological examination, vital signs assessment, and routine laboratory investigations, including complete blood count, coagulation profile, renal and liver function tests, and inflammatory markers, performed using standard automated analyzers in the hospital laboratory according to manufacturer specifications and internal quality control procedures.

Neuroimaging was performed using standard institutional protocols. CT venography was conducted using a multidetector CT scanner with intravenous iodinated contrast, acquiring axial images with multiplanar reconstructions. MRI examinations were performed on a 1.5-Tesla system and included standard sequences (T1-weighted, T2-weighted, fluid-attenuated inversion recovery (FLAIR), and diffusion-weighted imaging), with MRV obtained using time-of-flight and/or contrast-enhanced techniques as clinically indicated. All imaging studies were independently reviewed and reported by experienced consultant radiologists.

The diagnosis of CVST was based on established diagnostic criteria, requiring compatible clinical features in conjunction with radiological evidence of cerebral venous thrombosis on neuroimaging. Only patients with radiologically confirmed CVST were included in the study. Exclusion criteria included patients without radiological confirmation of CVST, those with incomplete or insufficient medical records, and patients with alternative major neurological conditions that could mimic CVST, such as brain tumors or central nervous system infections (e.g., meningitis).

A pre-prepared data collection sheet was used to gather the data for the following variables: demographic data (age, gender, residency, and ethnicity), medical history and comorbidities (date of diagnosis, comorbidities, and any previous history of thrombotic events), clinical presentation (primary symptoms at the time of presentation and the duration of symptoms before diagnosis), risk factors (oral contraceptive use, pregnancy/postpartum status, recent surgery, trauma, infection before CVST diagnosis, prolonged immobility, lifestyle factors such as smoking and obesity, and any positive family history of thrombotic disorders), diagnostic imaging techniques and the site of thrombosis, challenges that delayed diagnosis (non-specific symptoms, delayed presentation, and limitations of imaging techniques), complications (immediate complications including intracranial hemorrhage, stroke, hydrocephalus, and seizures, and long-term complications including persistent headaches, cognitive deficits, motor deficits, and recurrent thrombotic events), and treatment protocol (anticoagulation therapy, surgical intervention, or symptomatic treatment, and the duration of treatment).

Participants’ anonymity and confidentiality were preserved throughout the investigation. Data were entered and analyzed using the Statistical Package for the Social Sciences (SPSS), version 26 (IBM Corp., Armonk, NY, US). Appropriate statistical tests were conducted: independent samples t-tests were used for continuous variables, and Chi-squared tests were used for categorical variables. The significance threshold (p-value) was set at ≤0.05 for all statistical analyses.

## Results

Table 1 summarizes the demographic characteristics, risk factors, clinical presentation, management, and outcomes of 21 patients with CVST. The cohort was predominantly female (61.9%) with a wide age range. Headache was the most common presenting symptom (71.4%), followed by visual disturbances and seizures (42.9% each). Oral contraceptive use and infection were the most frequently identified risk factors (33.3% each). The superior sagittal sinus was the most commonly affected site (42.9%). Delayed diagnosis was mainly attributed to non-specific symptoms and delayed presentation. Most patients received anticoagulation therapy (95.2%). Seizures were the most frequent immediate complication (47.6%), while persistent headache was the most common long-term outcome (61.9%).

**Table 1:** Demographic characteristics, risk factors, clinical features, management, and outcomes of patients with cerebral venous sinus thrombosis (n = 21) SD: standard deviation

Domain	Variable	Finding
Demographics	Age (years), mean ± SD (range)	37.0 ± 19.6 (14–95)
	Sex: male	8 (38.1%)
	Sex: female	13 (61.9%)
	Residency: rural	9 (42.9%)
	Residency: urban	12 (57.1%)
Risk factors	No identifiable risk factor	1 (4.8%)
	Smoking	5 (23.8%)
	Obesity	5 (23.8%)
	Oral contraceptive use	7 (33.3%)
	Pregnancy/postpartum period	4 (19.0%)
	Infection	7 (33.3%)
	Malignancy	1 (4.8%)
	Sickle cell anemia	1 (4.8%)
	Positive family history of thrombosis	3 (14.3%)
	Previous thrombotic events	4 (19.0%)
	Other comorbid conditions	9 (42.9%)
Clinical presentation	Duration of symptoms (days), mean ± SD (range)	13.47 ± 10.7 (1–150)
	Headache	15 (71.4%)
	Visual disturbances	9 (42.9%)
	Seizures	9 (42.9%)
	Focal neurological deficits	2 (9.5%)
	Altered consciousness	2 (9.5%)
Site of thrombosis	Superior sagittal sinus	9 (42.9%)
	Cavernous sinus	8 (38.1%)
	Transverse and sigmoid sinuses	4 (19.0%)
Factors contributing to delayed diagnosis	Non-specific clinical symptoms	12 (57.1%)
	Delayed presentation to medical care	11 (52.4%)
	Imaging limitations	9 (42.9%)
Management	Symptomatic treatment	21 (100%)
	Anticoagulation therapy	20 (95.2%)
	Antiseizure medications	5 (23.8%)
	Antibiotic therapy	1 (4.8%)
	Surgical intervention	1 (4.8%)
	Duration of treatment (days), mean ± SD (range)	9.71 ± 3.08 (5–14)
Immediate complications	Seizures	10 (47.6%)
	Intracerebral hemorrhage	4 (19.0%)
	Papilledema	4 (19.0%)
	Cranial nerve palsy	2 (9.5%)
	Blindness	1 (4.8%)
	Proptosis	1 (4.8%)
Long-term outcomes	Persistent headache	13 (61.9%)
	Seizures	6 (28.6%)
	Motor deficits	2 (9.5%)
	No long-term complications	3 (14.3%)

Table 2 presents data comparing types of sinus thrombosis (sagittal sinus, transverse/sigmoid sinus, and cavernous sinus) across age and gender variables. The mean age for each type of thrombosis is reported, with no significant differences in age (p-value = 0.131). Gender distribution shows a higher percentage of men in transverse/sigmoid sinus thrombosis (n = 3, 75%) compared to sagittal sinus (n = 2, 22.2%) and cavernous sinus thrombosis (n = 3, 37.5%), but the difference is not statistically significant (p-value = 0.195).

**Table 2:** Association between demographic characteristics and the type of sinus thrombosis SD: standard deviation

Variables	Sagittal sinus thrombosis	Transverse/sigmoid sinus thrombosis	Cavernous sinus thrombosis	p-value	χ²/F-value
Age (mean ± SD)	29.55 ± 12.19	53.25 ± 15.1	37.25 ± 17.2	0.131	2.280
Male	2 (22.2%)	3 (75.0%)	3 (37.5%)	0.195	3.283
Female	7 (77.8%)	1 (25.0%)	5 (62.5%)		

Table 3 compares the presence of various risk factors across types of sinus thrombosis. No significant trends were observed across the risk factors. Notably, "no identifiable risk factor" was observed in cavernous sinus thrombosis (n = 1, 25%), but the difference was not significant (p-value = 0.343). Smoking, obesity, oral contraceptive use, pregnancy/postpartum, infection, cancer, sickle cell anemia, family history of thrombotic disorders, previous thrombotic events, and other comorbidities showed similar distributions with no significant differences across the three types of thrombosis (p-values ranging from 0.107 to 0.989).

**Table 3:** Association between risk factors and the type of sinus thrombosis

Variables	Sagittal	Transverse/sigmoid	Cavernous	p-value	χ²-value
No identifiable risk factor	0	0	1 (25.0%)	0.343	1.706
Smoking	2 (22.2%)	1 (25.0%)	2 (25.0%)	0.989	0.022
Obesity	2 (22.2%)	2 (50.0%)	2 (25.0%)	0.764	1.128
Oral contraceptive use	3 (33.3%)	1 (25.0%)	3 (37.5%)	0.911	0.188
Pregnancy/postpartum	2 (22.2%)	0	2 (25.0%)	0.553	1.184
Infection	4 (44.4%)	1 (25.0%)	2 (25.0%)	0.646	0.875
Cancer	0	1 (25.0%)	0	0.107	4.462
Sickle cell anemia	1 (11.1%)	0	0	0.231	1.400
Positive family history	1 (11.1%)	0	2 (25.0%)	0.475	1.491
Previous thrombotic events	1 (11.1%)	1 (25.0%)	2 (25.0%)	0.671	0.643
Other comorbidities	3 (33.3%)	3 (75.0%)	3 (37.5%)	0.347	2.115

Table 4 examines the clinical features and duration of symptoms for different types of sinus thrombosis. The most common symptom across all groups was headache, with 75% (n = 3) of cavernous sinus thrombosis patients and 33.3%-75% of others affected, though the difference was not statistically significant (p-value = 0.916). Visual disturbances were common, particularly in sagittal sinus thrombosis (n = 5, 55.6%) and transverse/sigmoid sinus thrombosis (n = 2, 50%). Focal neurological deficits and altered consciousness were more prevalent in cavernous sinus thrombosis, but these were not statistically significant (p-values = 0.166 and 0.166). The duration of symptoms ranged from 5.75 to 20.9 days, with no significant difference between groups (p-value = 0.640).

**Table 4:** Association between clinical features and the type of sinus thrombosis SD: standard deviation

Variables	Sagittal	Transverse/sigmoid	Cavernous	p-value	χ²/F-value
Headache	3 (33.3%)	3 (75.0%)	6 (75.0%)	0.916	3.646
Visual disturbances	5 (55.6%)	2 (50.0%)	2 (25.0%)	0.424	1.718
Focal neurological deficits	0	0	2 (25.0%)	0.166	3.592
Seizures	6 (66.7%)	4 (50.0%)	0	0.084	0.583
Altered consciousness	0	0	2 (25.0%)	0.166	3.592
Duration of symptoms (days, mean ± SD)	20.9 ± 16.16	12.25 ± 10.7	5.75 ± 3.19	0.640	0.460

Table 5 outlines the challenges delaying the diagnosis of sinus thrombosis across different types. Delayed presentation was more common in transverse/sigmoid sinus thrombosis (n = 21, 100%) and sagittal sinus thrombosis (n = 5, 55.6%), with a significant difference observed (p-value = 0.048). Non-specific symptoms were noted in 66.7% (n = 6) of sagittal sinus thrombosis cases and 50% (n = 2) in both transverse/sigmoid and cavernous sinus thrombosis, but this difference was not statistically significant (p-value = 0.747). Limitations of imaging techniques were reported in 44.4% (n = 4) of sagittal sinus thrombosis cases and 50% (n = 4) in cavernous sinus thrombosis, with no significant difference across the groups (p-value = 0.706).

**Table 5:** Association between challenges in diagnosis and the type of sinus thrombosis

Challenges	Sagittal	Transverse/sigmoid	Cavernous	p-value	χ²-value
Delayed presentation	5 (55.6%)	4 (100.0%)	2 (25.0%)	0.048	6.077
Non-specific symptoms	6 (66.7%)	2 (50.0%)	4 (50.0%)	0.747	0.583
Limitations of imaging	4 (44.4%)	1 (25.0%)	4 (50.0%)	0.706	0.697

Table 6 presents immediate and long-term complications across different types of sinus thrombosis. For immediate complications, acute symptomatic seizures were most common in sagittal sinus thrombosis (n = 6, 66.7%) and transverse sinus thrombosis (50%), with no significant difference between groups (p-value = 0.084). ICH was reported in 33.3% (n = 3) of sagittal sinus thrombosis cases, with no significant difference across groups (p-value = 0.830). Long-term complications show that persistent headache was common in all types of thrombosis, with 66.7% (n = 6) of sagittal sinus and 62.5% (n = 5) of cavernous sinus thrombosis cases affected, but no significant differences (p-value = 0.849). Seizures were more prevalent in sagittal sinus thrombosis (n = 5, 55.6%) compared to other groups (p-value = 0.05), indicating a statistically significant difference.

**Table 6:** Association between complications and the type of sinus thrombosis ICH: intracerebral hemorrhage

Variables	Sagittal	Transverse/sigmoid	Cavernous	p-value	χ²-value
ICH	3 (33.3%)	1 (25.0%)	2 (25.0%)	0.830	0.175
Seizures (immediate)	6 (66.7%)	4 (50.0%)	0	0.084	4.964
Papilledema	1 (11.1%)	2 (50.0%)	1 (12.5%)	0.215	3.075
Blindness	0	1 (25.0%)	0	0.107	4.462
Proptosis	1 (11.1%)	0	0	0.497	1.400
Cranial nerve palsy	1 (11.1%)	0	1 (12.5%)	0.655	0.530
Persistent headache	6 (66.7%)	2 (50.0%)	5 (62.5%)	0.849	0.328
Motor deficit	0	1 (25.0%)	1 (12.5%)	0.343	2.141
Remote seizures (epilepsy)	5 (55.6%)	0	1 (12.5%)	0.05	5.824
No complications	0	2 (50.0%)	1 (12.5%)	0.01	5.688

## Discussion

CVST is a rare but serious condition that can lead to significant morbidity and mortality if not promptly diagnosed and treated. It is characterized by thrombosis within the cerebral venous system, which can result in increased intracranial pressure, venous infarctions, and hemorrhages [9]. The present study aimed to analyze the demographic characteristics, risk factors, clinical presentation, site of thrombosis, challenges in diagnosis and treatment, and complications associated with CVST.

The study findings demonstrated a higher prevalence of CVST among women (n = 13, 61.9%), consistent with previous reports showing a female predominance [10]. This observation is commonly attributed to sex-specific risk factors, particularly hormonal influences such as oral contraceptive use and pregnancy or the postpartum period [11,12]. In the present cohort, the most frequently identified risk factors were oral contraceptive use (n = 7, 33.3%), infections (n = 7, 33.3%), smoking (n = 5, 23.8%), and obesity (n = 5, 23.8%), aligning with earlier studies that reported comparable risk factor profiles [13,14]. These findings reinforce the role of potentially modifiable risk factors in the pathogenesis of CVST. In contrast to studies in which inherited or acquired thrombophilic disorders represent the predominant underlying etiology [15], a relatively small proportion of patients in this cohort had a positive family history of thrombotic disorders (14.3%, n = 3) or a history of previous thrombotic events (19.0%, n = 4). This difference may reflect population-specific genetic factors, variations in environmental exposures, or limitations in the availability and routine use of comprehensive thrombophilia screening. The latter is particularly relevant in resource-limited settings and may contribute to underrecognition of underlying prothrombotic states, highlighting the need for improved diagnostic strategies and tailored risk assessment.

Headache was the most frequently reported presenting symptom in this study (n = 15, 71.4%), followed by seizures and visual disturbances (n = 9, 42.9% each). This pattern is consistent with previous studies identifying headache as the predominant symptom in CVST [16,17], reflecting the role of raised intracranial pressure and venous congestion in disease presentation. Notably, the mean duration of symptoms prior to diagnosis was 13.47 ± 10.7 days, highlighting a clinically significant diagnostic delay. This delay was largely attributable to non-specific symptomatology (n = 12, 57.1%) and delayed healthcare presentation (n = 11, 52.4%). Similar diagnostic challenges have been reported in the literature, with delayed presentation documented in up to 25% of cases [18,19]. These findings underscore the importance of maintaining a high index of clinical suspicion, particularly in patients presenting with persistent or atypical headaches, to facilitate earlier diagnosis and timely initiation of appropriate management.

Regarding the anatomical distribution of thrombosis, sagittal sinus involvement was the most frequently observed (n = 9, 42.9%), followed by cavernous sinus thrombosis (n = 8, 38.1%) and transverse/sigmoid sinus thrombosis (n = 4, 19.0%). This distribution is consistent with previous studies reporting the superior sagittal sinus as the most commonly affected site, followed by the cavernous sinus [20]. The observed variability in venous sinus involvement may reflect differences in underlying risk factors, such as infection-related cases predisposing to cavernous sinus thrombosis, as well as demographic and population-specific characteristics. These findings highlight the heterogeneity of CVST and underscore the importance of comprehensive neuroimaging to accurately delineate the extent and location of thrombosis.

The treatment approach in this cohort was primarily based on symptomatic management (n = 21, 100%) and anticoagulation therapy (n = 20, 95.2%), in accordance with current guidelines that recommend anticoagulation as the cornerstone of CVST management, even in the presence of intracranial hemorrhage [21]. The relatively limited use of antiseizure medications (n = 5, 23.8%) and surgical intervention (n = 1, 4.8%) reflects the heterogeneity of disease severity and complication burden within the cohort. These findings suggest that, while most patients can be effectively managed with medical therapy alone, a subset may require additional targeted interventions depending on clinical presentation and the development of complications [4,22].

Immediate complications observed in this cohort included seizures (n = 10, 47.6%), intracranial hemorrhage (n = 4, 19.0%), and papilledema (n = 4, 19.0%), all of which are well-recognized complications of CVST and consistent with existing literature [20,22]. The high frequency of seizures may reflect cortical involvement and delayed diagnosis in a subset of patients. Long-term complications were also substantial, with persistent headache (n = 13, 61.9%) and epilepsy (n = 6, 28.6%) being the most commonly reported outcomes [23]. The observed burden of long-term morbidity highlights that CVST is not invariably a benign condition and underscores the need for structured long-term follow-up, multidisciplinary care, and rehabilitation strategies to optimize functional recovery and quality of life.

Age and gender distributions did not demonstrate statistically significant differences across sinus thrombosis subtypes (p > 0.05). Nevertheless, a trend toward older age was observed in patients with transverse and sigmoid sinus thrombosis (mean age 53.25 ± 15.1 years) compared with those with sagittal (29.55 ± 12.19 years) and cavernous (37.25 ± 17.2 years) sinus thrombosis. This observation is consistent with previous reports suggesting that age may influence the anatomical distribution of CVST [24]. Younger patients are more likely to develop sagittal and cavernous sinus thrombosis, potentially due to hormonal factors such as oral contraceptive use and pregnancy, as well as a higher prevalence of infection-related cases leading to cavernous sinus involvement. In contrast, transverse and sigmoid sinus thrombosis appears more frequently in older individuals, possibly reflecting an increased burden of systemic prothrombotic conditions, including malignancy and cardiovascular disease. Variations in venous drainage patterns and age-related changes in coagulation dynamics may further contribute to these observed differences [12].

Risk factor profiles varied according to the anatomical site of thrombosis. Oral contraceptive use was frequently observed in cases of sagittal sinus thrombosis (n = 3, 33.3%) and cavernous sinus thrombosis (n = 3, 37.5%), while it was less common in transverse sinus thrombosis (n = 1, 25%). Similarly, pregnancy and postpartum states were associated with sagittal and cavernous sinus involvement but were not observed in transverse sinus thrombosis, a pattern consistent with the established influence of hormonal factors on CVST risk [12,25]. Infection was more frequently identified in sagittal sinus thrombosis (n = 4, 44.4%) compared with cavernous (n = 2, 25%) and transverse (n = 1, 25%) sinus thrombosis. Although cavernous sinus thrombosis is classically linked to facial and orbital infections, the predominance of infection-related cases in sagittal sinus thrombosis in this cohort may reflect regional patterns of infectious disease or delays in diagnosis, underscoring the heterogeneity of CVST etiologies across different venous sinuses [26].

Headache was the most frequently reported symptom across all thrombosis subtypes, with the highest prevalence observed in cavernous sinus thrombosis (n = 6, 75.0%) and the lowest in sagittal sinus thrombosis (n = 3, 33.3%), a distribution consistent with previous studies [27]. Seizures were more commonly observed in sagittal sinus thrombosis (n = 6, 66.7%) compared with transverse sinus thrombosis (n = 4, 50%) and were not reported in cavernous sinus thrombosis, although this difference did not reach statistical significance (p = 0.084). This pattern aligns with existing evidence suggesting an increased seizure risk in superior sagittal sinus thrombosis due to cortical vein involvement and associated parenchymal irritation [28].

Delayed diagnosis was more frequently observed in transverse sinus thrombosis, affecting all cases in this subgroup (n = 4, 100%), compared with sagittal sinus thrombosis (55.6%) and cavernous sinus thrombosis (25%), with this difference reaching statistical significance (p = 0.048). This finding is consistent with reports from recent studies [19] and likely reflects the relatively non-specific clinical presentation of transverse sinus thrombosis, which can mimic primary headache disorders or other benign conditions, thereby complicating early recognition and contributing to diagnostic delay.

We observed ICH in 25.0% (n = 2) of cases of cavernous and transverse sinus thrombosis, but it was slightly higher in sagittal sinus thrombosis (n = 3, 33.3%). This is due to increased venous pressure, which leads to venous congestion, rupture of fragile cortical veins, and blood-brain barrier disruption. The sagittal sinus is particularly prone due to its extensive cortical venous drainage, while cavernous sinus thrombosis can cause retrograde venous hypertension affecting deep venous structures. These hemorrhagic complications contribute to neurological deterioration and worse clinical outcomes [16]. Long-term complications were most notable in sagittal sinus thrombosis, with persistent headache occurring in 66.7% (n = 6) of cases. The high rate of persistent headaches in all types of thrombosis suggests that they have a big effect on quality of life. This is in line with previous research [17], which found that intracranial hypertension occurred in about 10% of people after CVST. Insufficient recanalization status may facilitate this, and patients with visual disturbances seem to develop intracranial hypertension later. Those patients who present with intracranial hypertension after CVST may develop recurrent CVST. Although causal relationships cannot be established, delayed diagnosis and underlying etiology appear to influence long-term outcomes in CVST. Prolonged symptom duration before diagnosis may increase the risk of persistent headache and epilepsy, while certain etiologies, such as prothrombotic states and infection-related CVST, may predispose patients to sustained neurological sequelae.

This study provides novel data on CVST from Iraq, a region where published evidence is currently lacking. Unlike large international cohorts such as the International Study on Cerebral Vein and Dural Sinus Thrombosis (ISCVT), which predominantly represent high-income and well-resourced healthcare systems, the present study reflects real-world practice in a resource-limited setting. The absence of prior Iraqi data and the scarcity of regional reports from Arab countries highlight the importance of this cohort. Our findings reveal similarities with international data in terms of female predominance and headache as the most common presenting symptom, while also emphasizing context-specific challenges, including delayed presentation, non-specific symptoms, and limited access to advanced imaging. These factors may contribute to diagnostic delays and influence clinical outcomes. By addressing this important geographical gap, this study adds region-specific evidence that may inform local clinical decision-making and serve as a foundation for future national and regional multicenter studies.

Despite its valuable insights, this study has several limitations. The sample size was relatively small (n = 21), which limits the generalizability of the findings and precludes meaningful subgroup analyses or multiple comparisons. Owing to the limited sample size, multivariable analysis was not feasible, and this should be considered when interpreting associations between clinical variables and outcomes. The retrospective nature of data collection introduces the potential for selection bias and incomplete medical records. In addition, the absence of routine thrombophilia screening in all patients may have resulted in the underestimation of hereditary prothrombotic conditions. The single-center design further limits the applicability of the results to broader populations. From a public health perspective, these limitations underscore the need for improved clinician awareness of CVST, particularly in low-resource settings where non-specific presentations and delayed referrals are common. Enhanced access to timely and advanced neuroimaging, along with the development of standardized diagnostic pathways, may help reduce diagnostic delays and improve outcomes in similar healthcare contexts.

## Conclusions

CVST predominantly affects young and female individuals, with the sagittal and cavernous sinuses being the most commonly involved sites. Major risk factors include oral contraceptive use, infections, pregnancy/postpartum state, obesity, and smoking. Headache is the most common presenting symptom, with seizures and visual disturbances also frequently reported. Diagnostic delays occur due to non-specific symptoms and limitations in imaging techniques, particularly for transverse sinus thrombosis. The majority of patients receive anticoagulation therapy, but symptomatic management is also essential. Immediate complications include seizures and ICH, while long-term complications primarily involve persistent headaches and recurrent seizures.

Early recognition of CVST should be emphasized, especially in young patients with risk factors such as oral contraceptive pill (OCP) use and infections. Clinicians should maintain a high index of suspicion for CVST in patients presenting with persistent headaches in the presence of risk factors. Improved imaging techniques and standardized protocols are needed to reduce diagnostic delays. Long-term follow-up is necessary to monitor persistent symptoms and prevent recurrent thrombotic events.
